# Recent advancements to study flowering time in almond and other *Prunus* species

**DOI:** 10.3389/fpls.2014.00334

**Published:** 2014-07-11

**Authors:** Raquel Sánchez-Pérez, Jorge Del Cueto, Federico Dicenta, Pedro Martínez-Gómez

**Affiliations:** ^1^Plant Biochemistry Laboratory, Department of Plant and Environmental Sciences, Faculty of Science, University of CopenhagenCopenhagen, Denmark; ^2^Department of Plant Breeding, CEBAS-CSICMurcia, Spain

**Keywords:** *Prunus dulcis*, breeding, almond, flowering time, dormancy, genome, transcription factors, molecular markers

## Abstract

Flowering time is an important agronomic trait in almond since it is decisive to avoid the late frosts that affect production in early flowering cultivars. Evaluation of this complex trait is a long process because of the prolonged juvenile period of trees and the influence of environmental conditions affecting gene expression year by year. Consequently, flowering time has to be studied for several years to have statistical significant results. This trait is the result of the interaction between chilling and heat requirements. Flowering time is a polygenic trait with high heritability, although a major gene *Late blooming* (*Lb*) was described in “Tardy Nonpareil.” Molecular studies at DNA level confirmed this polygenic nature identifying several genome regions (Quantitative Trait Loci, QTL) involved. Studies about regulation of gene expression are scarcer although several transcription factors have been described as responsible for flowering time. From the metabolomic point of view, the integrated analysis of the mechanisms of accumulation of cyanogenic glucosides and flowering regulation through transcription factors open new possibilities in the analysis of this complex trait in almond and in other *Prunus* species (apricot, cherry, peach, plum). New opportunities are arising from the integration of recent advancements including phenotypic, genetic, genomic, transcriptomic, and metabolomics studies from the beginning of dormancy until flowering.

## Introduction

From a commercial point of view, flowering time is one of the most important agronomic traits in almond (*Prunus dulcis* (Miller) D. A. Webb) as it determines the vulnerability of production to late frosts, as well as the use of cultivars for cross-pollination in order to achieve successful pollination when the flowering time of two varieties must coincide (Dicenta et al., 2005).

Breeding new, late flowering almond cultivars is a very long and costly task since, due to the long juvenile period, their first flowering usually occurs in the third year after plantation in the field, or even later. In addition, the influence of climatic factors on this trait obligates the breeder to record the data for several years (Dicenta et al., 1993, 2005). In this sense, it would be very useful to have tools for early selection of the latest flowering seedlings in the nursery (after germination of seeds), which would be planted in the experimental orchards for further selection (Dicenta et al., 2005). Flowering will only happen when dormancy is broken. Endodormancy has been described as the inability of a tree to start floral or vegetative budbreak, even with moderate temperatures. Endodormancy occurs prior to ecodormancy, which happens in late winter and spring and is imposed by temperatures unfavorable to growth (Sánchez-Pérez et al., 2012).

On the other hand, almond and other *Prunus* species (apricot, cherry, peach, plum, etc.) accumulate a mono cyanogenic glucoside (CNGLc) called prunasin in different vegetative and reproductive parts of the plant, and in the seeds a di-CNGLc called amygdalin (McCarty et al., 1952; Frehner et al., 1990; Sánchez-Pérez et al., 2008). When specific enzymes called β-glucosidases degrade CNGLcs, glucose, benzaldehyde and cyanide are released (Morant et al., 2008). Upon degradation of hydrogen cyanamide—a nitrogen-based chemical compound sprayed in the flower buds—nitroxil and cyanide are released. This brings forward the flowering time in apple, apricot, peach, persimmon, sweet cherry flower buds, etc., when compared to untreated plants (Dozier et al., 1990; George et al., 1992; Faust et al., 1997).

In this work, recent advancements to study flowering time in almond have been included at the phenotypic (observed trait), genetic (inheritance and transmission), genomic (DNA analysis), transcriptomic (gene expression analysis) and metabolomic (metabolites involved, as cyanide content) level.

## Phenotypic studies

In general, the accuracy of phenotypic evaluation is critical for further reliable genetic and molecular studies. To diminish the significant influence of the environment, flowering time can be dissected as the sum of two traits: chilling and heat requirements. These can be determined in monitored conditions by measuring the temperatures in the field and simultaneously controlling temperatures and humidity in the growth chamber (Egea et al., 2003; Sánchez-Pérez et al., 2012). Chill requirements have a much stronger effect on flowering time compared to heat requirements (Egea et al., 2003) with a high positive correlation between chilling requirements and flowering time (Sánchez-Pérez et al., 2012).

These results were also verified in other *Prunus* species such as apricot (*P. armeniaca* L.) (Ruiz et al., 2007) and sweet cherry (*P. avium* L.) (Alburquerque et al., 2008). However, Alonso et al. (2005) described a higher influence of heat requirements than chilling requirements on the flowering time in cold areas, using a mathematical model recording temperatures and flowering time, but not evaluating the endodormancy breaking in flower buds.

## Genetic studies

Inheritance studies of the genetic control of flowering time on almond showed that flowering time is a polygenic trait (Kester et al., 1977; Dicenta et al., 1993; Sánchez-Pérez et al., 2007a). However, a major dominant gene controlling this trait was described specifically in some descendants of the almond cultivar “Tardy Nonpareil,” considered a late flowering mutant of “Nonpareil” (Kester, 1965; Socias i Company et al., 1999; Sánchez-Pérez et al., 2007a). These authors, studying some descendants of “Tardy Nonpareil,” observed a bimodal distribution for this trait, which was explained by the presence of a late blooming major gene (*late blooming*, *Lb*), quantitatively modified by minor genes.

Flowering time of almond has a high heritability (Kester et al., 1977; Dicenta et al., 1993), so crossing late-flowering parents will produce late-flowering seedlings (Dicenta et al., 2005). The best breeding strategy to obtain late-blooming almond descendants is therefore to cross parents as late-blooming as possible, and when the offspring shows a bimodal distribution, the latest-blooming genotypes should be selected, probably carrying the *Lb* allele, which could be transmitted to its descendants (Sánchez-Pérez et al., 2012).

On the other hand, studies on the genetic basis and inheritance of chilling and heat requirements to break endodormany and ecodormancy in almond are scarcer. Sánchez-Pérez et al. (2012) described a polygenic nature of these traits in accordance with the observed flowering time of seedlings. In addition, these authors observed, in a 2-year study, that the bimodal distribution of chilling requirements in the studied progeny could be explained by the presence of the *Lb* gene hypothetically linked to these chilling requirements. In other *Prunus*, quantitative inheritance of chilling requirements for breaking endodormancy was also observed in flower buds in peach (Fan et al., 2010) and vegetative buds in apricot (Olukolu et al., 2009). In this species, a higher influence of chilling requirements on flowering time was also described, although a single recessive gene called *EVERGROWING (EVG)* gene (Rodríguez et al., 1994) was described as a chilling and heat requirement related gene (Bielenberg et al., 2004, 2008). Interestingly, the deletion of four out of six of the *Dormancy Associated MADS-box* (*DAM1-DAM6*) genes in the *evg* mutant caused the transcriptional inhibition of the other two structurally intact genes of the family (Bielenberg et al., 2008), in which no flowering occurred.

More is known about the genetic bases of flowering time in the long day plant *Arabidopsis thaliana* than *Prunus* species. More than 60 genes were identified as regulating flowering time in *Arabidopsis* (Ehrenreich et al., 2009), and due to SNPs present in genes including *CONSTANS* (*CO*), *FLOWERING LOCUS C* (*FLC*), *VERNALIZATION INSENSITIVE 3*, *PHYTOCHROME D*, *GIBBERELLIN*, etc. or coding region deletions as in *FRIGIDA* gene, there are different phenotypes for flowering time in *Arabidopsis*. In fact, dominant alleles of *FRI* generate late flowering phenotypes while *FRI* mutants are early flowering (Johanson et al., 2000; Jung and Müller, 2009). Early flowering is also possible if *APETALA1* (*AP1*) is constitutively expressed (Chi et al., 2011). When *PsAP1*, *AP1* from cherry (*Prunus avium* L.), was over-expressed in *Arabidopsis*, it produced an early flowering phenotype, shortening the juvenile period (Wang et al., 2013). *AP1* belongs to the *MADS-box* gene family. *MdMADS5*, an *AP1*-like gene of apple (*Malus* × *domestica*), also caused early flowering in transgenic *Arabidopsis* (Kotoda et al., 2002), as did *MdMADS2* in transgenic tobacco (*Nicotiana tabacum*). *AP1* is activated by *LEAFY* (*LFY*), which is also involved in flowering time in *Arabidopsis*. Moreover, *TERMINAL FLOWER 1* (*TFL1*) and *FLOWERING LOCUS T* (*FT*) are two key regulators of flowering time. These genes belong to the PEBP family but they have antagonistic functions. *TFL1* interacts with b-ZIP transcription factor *FD* and represses the transcription of *FD*-dependent genes as *AP1* and *AGAMOUS* (*AG*), while *FT* interacting with *FD* activates *AP1* and *AG* (Wang and Pijut, 2013). *TFL1* homologous genes such as *PsTFL1* (*P. serotina* Ehrh., *TFL1* homolog) delay flowering when expressed in *Arabidopsis*. Homologous genes from *Arabidopsis* found in *Prunus* species are shown in Table 1.

**Table 1:** **Identified genes involved in the regulation of flowering time in *Prunus* species**.

**Specie**	**Gene**	**Gene ID**	**Annotation**	**References**
*P. armeniaca*	*PHYE*	Q6SCK5	Phytochrome E	GeneBank data
*P. armeniaca*	*RGA*	RGA	Transcription factor	Soriano et al., 2005
*P. armeniaca*	*SOC1*	L7Y228	Transcription factor	Trainin et al., 2013
*P. armeniaca*	*TFL1*	ADL62862	Phosphatidylethanolamine binding	Liang et al., 2010
*P. avium*	*APETALA1*	APETALA1	MADS-box gene family	Wang et al., 2013
*P. avium*	*GA1*	GA1	Gibberellin biosynthesis	Blake et al., 2000
*P. avium*	*TFL1*	AB636121.1	Phosphatidylethanolamine binding	Mimida et al., 2012
*P. domestica*	*FT1*	FT1	Transcription coactivator	Tränkner et al., 2010
*P. dulcis*	*GA20*	BU574794	Gibberellin 20-oxidase	Silva et al., 2005
*P. dulcis*	*LFY*	PrdLFY	AFL2	Silva et al., 2005
*P. dulcis*	*MADS1*	PrdMADS1	MdMADS10	Silva et al., 2005
*P. dulcis*	*PHYA*	Q94EK7	Phytochrome A	GeneBank data
*P. dulcis*	*TFL*	BU574411	Flowering locus T-like protein	Silva et al., 2005
*P. mume*	*AGL 24*	AB437345.1	dormancy-associated MADS-box	Yamane et al., 2008
*P. mume*	*FT*	AM943979	Flowering locus T protein	GeneBank data
*P. serotina*	*AGAMOUS*	EU938540	Formation of stamens and carpels	Liu et al., 2010
*P. persica*	*AGL 2*	BU048398	MdMADS 8	Silva et al., 2005
*P. persica*	*AP1*	BU039475	MdMADS 2	Silva et al., 2005
*P. persica*	*AP2*	BU046298	AHAP2	Silva et al., 2005
*P. persica*	*ATM YB33*	XM_007218900	myb domain protein 33	Zhu et al., 2012
*P. persica*	*CDF1*	XM_007215192	cycling DOF factor 2	GeneBank data
*P. persica*	*CO*	BU042239	Constans like protein	Silva et al., 2005
*P. persica*	*DAM 5*	AB932551	MADS-box 5	Yamane et al., 2011
*P. persica*	*DAM 6*	DQ863252	MADS-box 6	Bielenberg et al., 2008
*P. persica*	*FAR 1*	BU047045	Far-red-impaired responsive protein	Silva et al., 2005
*P. persica*	*FPF 1*	XM_007225892	Flowering promoting factor	Romeu et al., 2014
*P. persica*	*FRL 1*	DY640223.1	FRI-related gene	Silva et al., 2005
*P. persica*	*FT*	G3GAW0	Flowering locus T protein	GeneBank data
*P. persica*	*FT*	BU044758	Flowering locus T protein	Silva et al., 2005
*P. persica*	*LEAFY*	EF175869	Activator of AP1	An et al., 2012
*P. persica*	*PHYA*	Q945F7	Phytochrome A	GeneBank data
*P. persica*	*PHYB*	Q945T4	Phytochrome B	GeneBank data
*P. persica*	*TFL1*	ADL62867	Phosphatidylethanolamine binding	Liang et al., 2010

On the other hand, the adaptation of the short day model plant rice (*Oryza sativa* L.) to different climate conditions depends on variation in flowering time, also called “heading date (hd).” A number of genes and QTLs have been identified in rice, including *Hd1*/*Se1*, *Ehd1*, *Hd3a*, and *RFT1* (*Rice Flowering Locus T1*) among others (Ogiso-Tanaka et al., 2013). Functional defects in *RFT1* are the main reasons for late flowering rice in an *indica* cultivar. *Hd3, RFT1*, and *FT* are florigen genes. FT protein is the long-sought florigen that activates the transcription of FD, forming the florigen activation complex (FAC). FAC will bind to the promoter regions of floral meristem identity genes such as e.g., *AP1*, so flowering will occur (Taoka et al., 2013). Therefore, flowering time is affected by florigen in *Arabidopsis* and rice.

## Genomic studies

**Figure 1:**
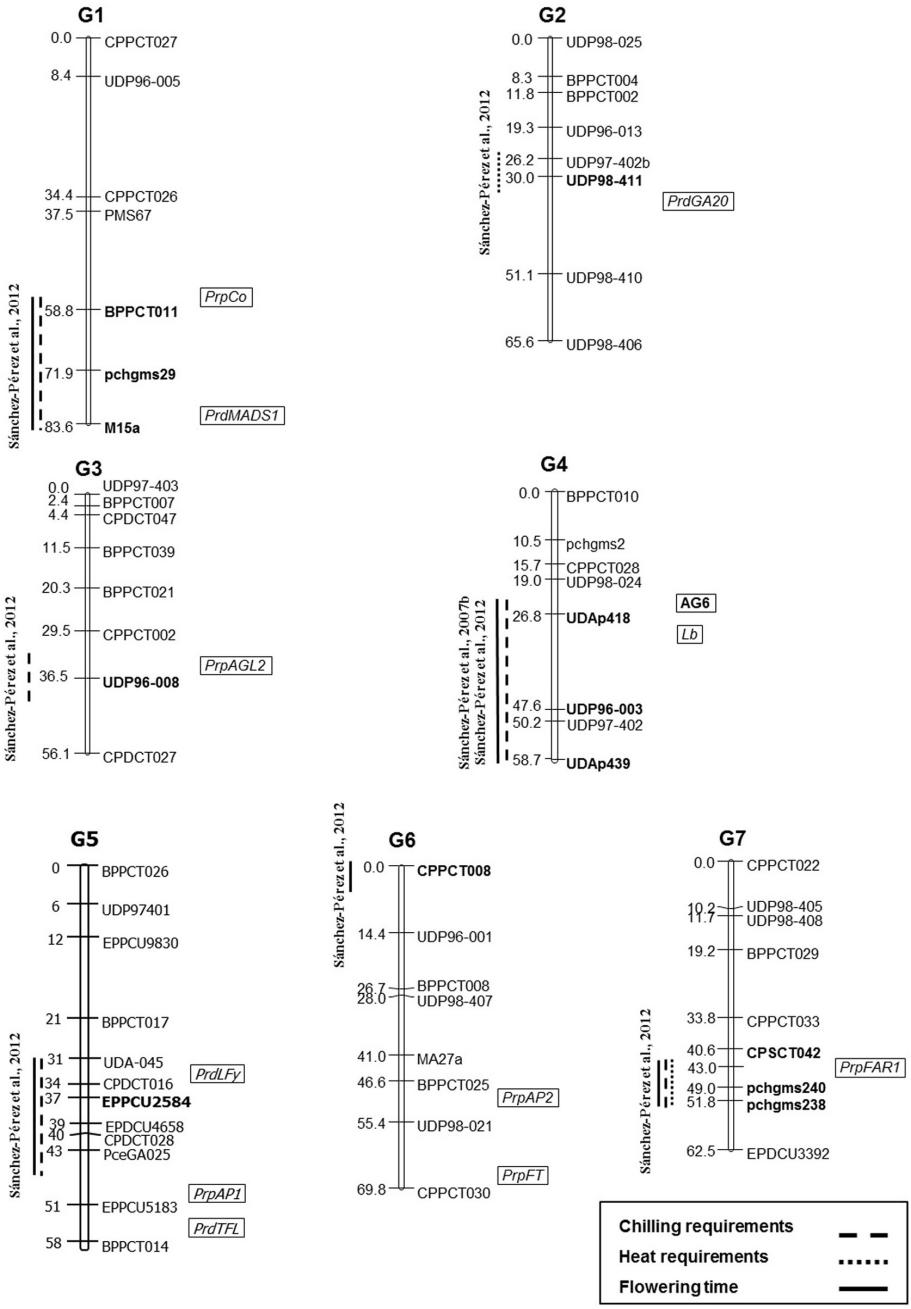
**Location of QTLs linked to flowering time and chilling and heat requirements in the almond map from the population R1000 × Desmayo Largueta performed by Sánchez-Pérez et al. (2007b, 2012)**. The closest SSR marker linked to the QTL is marked with bold lettering on this map. The approximately location of the RAPD marker (AG6) (Ballester et al., 2001) and candidate genes (*in italics*) (Silva et al., 2005) linked to flowering time in other almond populations, are indicated inside the boxes. The integration of this information from different genetic maps has been performed using the centimorgan (cM) distances indicated by the different authors in each linkage group and each map.

The first genomic studies performed used RAPDs (Random Amplified Polymorphic DNA) and bulk segregant analysis in a F1 progeny from “Tardy Nonpareil,” corroborating the presence of the previously mentioned major gene *Lb* controlling late flowering time. Moreover, three RAPDs were found to be associated with *Lb* in linkage group 4 (G4) of the “Felisia” × “Bertina” (“Felisia” is a descendant from “Titan,” that is a seedling of “Tardy Nonpareil”) genetic map (Ballester et al., 2001). In addition, Silva et al. (2005) described several Quantitative Trait Loci (QTLs) linked to flowering time in an interspecific F1 almond × peach progeny using a Candidate Gene approach (CG) in G1, G2, G3, G5, G6, and G7. More recently, different works using SSR (Simple Sequence Repeat) markers in a F1 population between a seedling of “Tardy Nonpareil” (“R1000”) × “Desmayo Largueta” (R×D), also confirmed the location of *Lb* in G4 and identified other QTLs to flowering time in G1, G6, and G7 (Sánchez-Pérez et al., 2007b; Martínez-Gómez et al., 2012) (Figure 1). In the first study (2007), carried out in this R×D population, the SSR UDP-96003 was located very close to the *Lb* gene in G4 of the map. When QTL analysis was performed, this major QTL (*Lb*) in G4 was able to explain between 56.5 and 86.3% of the variance in “R1000,” which is supposed to carry the *Lb* gene, and 54.5–67.7% of the variance in the mapped R×D population.

In other *Prunus* species, QTLs associated with *flowering time* were also described in peach, apricot and cherry, confirming the polygenic nature. In peach, Fan et al. (2010) described different QTLs linked to flowering time in G1, G2, G4, G6, G7, and G8. Campoy et al. (2011) and Salazar et al. (2013) described a QTL linked to flowering time in G1, G5, and G7 in apricot. Finally, Wang et al. (2000); Dirlewanger et al. (2012), and Castede et al. (2014) also identified a QTL linked to flowering time in G1, G2, and G4 of sour and sweet cherry.

Silva et al. (2005) and Sánchez-Pérez et al. (2012) described several QTLs linked to *chilling* (G1, G3, G4, G5, G6, and G7) and *heat* (G2 and G7) requirements in almond (Figure 1), confirming again its polygenic nature. Fan et al. (2010) also described several QTLs linked to chilling and heat requirements in G1, G2, G4, G6, G7, and G8 in peach. QTLs linked to chilling requirements of vegetative buds were described in apricot by Olukolu et al. (2009) in G1, G2, G3, G5, and G8. In sweet cherry (*P. avium* L.), Dirlewanger et al. (2012) and Castede et al. (2014) also found one QTL in G4 in sweet cherry.

Moreover, functionally characterized homologs from *Arabidopsis* were used as a CG approach with 10 genes from almond or peach (*PrdLFY*, *PrdMADS1*, *PrpAP1*, *PrpFT*, *PrpAGL2*, *PrpFAR1*, *PrdTFL*, *PrdGA20*, *PrpAP2*, and *PrpCO*) (Silva et al., 2005). These 10 genes were mapped in the *Prunus* reference map (Texas x Earlygold) and can be found in G1, G2, G3 G5, G6, and G7 (Figure 1, Table 1). However, none of them co-localized with *Lb* gene in G4. One reason for this is that there are more than 60 genes involved in flowering time in *Arabidopsis*, so other CGs should be analyzed. The other reason could be that the flowering time trait is due to different mechanisms in perennial plants than in annual plants.

The other recent CG analysis study was performed in an area of 3.7 Mbp in G4 (around the *Lb* gene) and showed 429 genes in the peach Lovell genome (Castede et al., 2014). Based on predicted function of proteins, they selected nine CGs. One of these, ppa002685m, is an *embryonic flower 2* gene involved in the vernalization response and a negative regulator of the flower development through histone methylation. It would be interesting to see the analysis of this gene in varieties with different flowering times in almond.

Recently, Zhebentyayeva et al. (2014) developed a comprehensive program to identify genetic pathways and potential epigenetic mechanisms involved in control of chilling requirement and flowering time in peach. These authors described the *TFL1*, which regulates the vegetative to reproductive transition, and the *PcG* (*polycomb group*) genes, which are involved in the epigenetic regulation of flowering in *Arabidopsis*. It is worth noting that these authors failed to identify a direct *FLC* gene ortholog and its regulator *FRI*, suggesting that control of flowering time in *Prunus* species has a complex genetic architecture.

The recent release of the complete peach genome sequence (Verde et al., 2013) together with four almond genome sequences (Koepke et al., 2013) and the sweet cherry genome publicly available since 2013 (Carrasco et al., 2013), offer new possibilities for integrating genetic and genomic approaches to find new CGs for flowering time in perennial plants (Martínez-Gómez et al., 2012).

## Transcriptomic studies

Almond transcriptomic studies have not been performed to date. The only transcriptomic study performed in other *Prunus* species has been using flower buds of Japanese apricot (*Prunus mume* Sieb. et Zucc.) at different dormant stages (Habu et al., 2012). In this species, varying flowering time is caused by irregular bud endodormancy release (Zhuang et al., 2013). The transcriptome analysis of flower buds showed 25 endodormant-specific up-regulated unigenes. *DAM6* was one of them although, in most of the unigenes, no hit was found. As we have previously mentioned, many of the *MADS* family genes, such as *DAM6*, are involved in different steps of flower development including flowering time (Riechmann and Meyerowitz, 1997). At this moment, more than *50 MADs-Type* Transcription Factors (TFs) have been identified in the peach genome, so further studies should be done to identify more gene products involved in flowering.

In *Arabidopsis*, flowering time is dependent on intricate genetic networks to perceive and integrate both endogenous and environmental signals (Khan et al., 2014). In the aging pathway, it has been found that the role of five microRNAs (miRNAs) families called *miR156*, *miR172*, *miR159/319*, *miR390*, and *miR399* is important in flowering time (Spanudakis and Jackson, 2014). Recently, miRNAs differently expressed in chilled peach vegetative buds have been identified co-localizing with known QTLs for chilling requirement and flowering time traits (Barakat et al., 2012; Ríos et al., 2014). A cascade of miRNAs such as miR156, miR172 and their respective targets *SQUAMOSA PROMOTER BINDING PROTEIN-LIKE*, and *AP2* like genes are involved in modulating flowering induction in *Arabidopsis* through *FT* and other flowering related genes (Khan et al., 2014; Spanudakis and Jackson, 2014). Transcriptomic studies in poplar and leafy spurge have shown a differential expression of *SPL* genes and *miR172* during dormancy induction, suggesting that this miRNA pathway may also play a regulating role in dormancy processes that affect flowering time (Ríos et al., 2014).

Application of new high-throughput RNA sequencing (RNA-seq) technologies (Flintoft, 2008; Martínez-Gómez et al., 2011) could greatly clarify the TFs involved in the regulation of flowering time, allowing the determination of transcripts from a particular region of the genome.

## Metabolomic studies

The common by-product upon degradation of hydrogen cyanamide and cyanogenic glucosides is cyanide, which is not only involved in bringing forward flowering time but also in breaking seed dormancy by inducing formation of Reactive Oxygen Species (ROS). ROS activates a cascade in which *ETHYLENE RESPONSE FACTOR 1* is implicated, producing germination-associated proteins (Oracz et al., 2007). CBF proteins belong to the *CBF*/*DRE* binding sub-family of the *APETALA2-ETHYLENE* responsive factor (Nakano et al., 2006). The action mechanism of nitrogen-based chemical treatments could involve the regulation of the effect of these TFs.

Further, in stone-fruit species (e.g., *Prunus* species), the presence of common regulatory mechanisms between the chilling requirements for seed and bud dormancy release have been suggested (Leida et al., 2012). Moreover, secondary metabolites as cyanogens were suggested to be involved in the germination of cocklebur seeds (*Xanthium pennsylvaniicum* Wallr.) in response to various nitrogenous compounds (Esashi et al., 1996). Other cyanogens such as the CNGlc prunasin have been described in flower parts in eucalyptus (*Eucalyptus cladocalyx* F. Muell.), seeing that young flower buds were the most cyanogenic, when reproductive organs were analyzed at various stages of development (Gleadow and Woodrow, 2000).

The integrated analysis of these well-known mechanisms reveals accumulation of cyanogenic glucosides and regulation of flowering time through TFs, which open new possibilities in the analysis of this complex trait in almond and the rest of *Prunus*.

## New perspectives

Almond is not only the earliest fruit tree to break dormancy but also shows the widest range of flowering time among all fruit and nut species (Socias i Company and Felipe, 1992), making it a suitable candidate for studying this important trait within perennial plants. There are many genes that are conserved during the evolution of flowering plants. However, there are other mechanisms i.e., miRNAs regulation or metabolite signaling, which could be also included in new studies to deepen the analysis of gene regulation of the flowering time in almond. The final objective continues to be the development of efficient molecular markers for selection in breeding programs. This would enable breeders to select the late flowering individuals in the nursery which would allow them to avoid yield losses due to frosts, which currently occurs in early flowering genotypes.

## Author contributions

Raquel Sánchez-Pérez and Pedro Martínez-Gómez participated in the coordination of the study. Federico Dicenta and Jorge Del Cueto collected and revised the genetic information. Pedro Martínez-Gómez and Raquel Sánchez-Pérez collected and revised genomic and transcriptomic information. Raquel Sánchez-Pérez and Jorge Del Cueto collected metabolomics information.

### Conflict of interest statement

The authors declare that the research was conducted in the absence of any commercial or financial relationships that could be construed as a potential conflict of interest.
